# Coupling of anticipation and breathing in expert flute performance: the influence of musical structure and practice

**DOI:** 10.3389/fcogn.2024.1425005

**Published:** 2024-09-17

**Authors:** Michel A. Cara, Divna Mitrovic

**Affiliations:** Music Institute, Pontificia Universidad Católica de Valparaíso, Valparaíso, Chile

**Keywords:** music reading, sight-reading, eye movement, eye-hand span, flute performance, breathing patterns, musical expertise, embodied music cognition

## Abstract

**Introduction:**

In this study, we examined the cognitive processes and physiological responses involved in learning a flute piece by the composer Charles Koechlin among musicians of different expertise levels. Participants performed the piece four times consecutively, with a 2-min practice interval between the first and the second trial.

**Methods:**

Using data obtained from an eye tracker, respiratory sensors, and an audio recorder we assessed short-term improvement and the effect of musical structure and practice on key variables identified through a multivariate approach: eye-hand span (EHS), time index of EHS, thoracic and abdominal amplitude (breathing patterns) and pupil dilation.

**Results:**

The analysis revealed two main dimensions: one associated with EHS; and the other with embodied responses to music, closely linked to breathing patterns and pupil dilation. We found an effect of musical structure on all the variables studied, while the EHS improved with practice. Expert musicians demonstrated enhanced EHS and adapted their breathing patterns more effectively to the music's structure.

**Discussion:**

These insights support the hypothesis of a coupling between anticipation and breathing, emphasizing the role of perceptual and embodied components in music reading and learning.

## 1 Introduction

The mechanisms of anticipation in musical reading have been investigated rather intermittently since the 1940s; however, little is known to date about the relationship between these mechanisms and the breathing patterns of wind instrument performers, which are fundamental elements in the planning of a musical interpretation. Our study aims to explore this relationship within the perspective of embodied musical cognition. This theoretical framework posits that the sensorimotor system is crucial for both the production and perception of music (Leman and Maes, 2015). Indeed, the reciprocal influence between movement and perception, along with the activation of motor areas of the brain during rhythm perception, are often considered indirect evidence for embodied musical cognition (Levitin et al., 2018). According to Matyja (2016), this metaphor, in which the human body serves as a mediator in the production of meaning, has been interpreted broadly and often somewhat ambiguously. Thus, current research should focus on exploring these physiological aspects of music response more deeply. Our study addresses this gap by utilizing three specific measures: breathing patterns, eye movements, and audio recordings of musical performances. These measures enable analysis of how anticipatory and respiratory mechanisms interact and influence musical interpretation, making physiological responses key aspects of our research.

Eye movements provide valuable insights into the perceptual and cognitive processes involved in music reading. Music reading is a complex task that requires the integration of multimodal information (Perra et al., 2021). It is associated with various perceptual and motor functions (Waters et al., 1997). Proficiency in music reading requires practice (Chang and Gauthier, 2021). Sight-reading, a crucial component, involves reading new music without prior rehearsal. This task necessitates the rapid translation of visual cues into motor actions, depending on working memory and anticipatory planning (Rosenbaum, 1991; Wurtz et al., 2009).

Anticipation in music is explored through the concept of the “Eye-Hand Span” (EHS), which measures the distance between the fixation point on the score and keypress data or the played note (Servant and Baccino, 1999; Rosemann et al., 2015). The EHS aligns eye movements with the musician's motor actions, offering insights into the strategies for playing individual notes (Perra et al., 2021). Weaver (1943) and Sloboda (1974, 1977) noted the variability and adaptability of the EHS to musical texture and structure. Furneaux and Land (1999) defined the EHS in terms of a “time index,” ranging from 0.5 s to 1.3 s and influenced by tempo. Recent studies show that the EHS is not significantly improved by short practice sessions (Rosemann et al., 2015; Cara, 2018), while others show improvement with practice (Burman and Booth, 2009; Cara, 2023). The EHS can be quantified using various units. It is affected by the complexity of the music score and some authors claim that it is not a decisive indicator of sight-reading competencies, but rather an anticipation regulation strategy (Lim et al., 2019). Most studies have focused on pianists, while a minority have examined singers or other instrumentalists. A study with xylophone players (Marandola, 2019), showed different gaze behavior strategies depending on cultural background, however the players' anticipation (eye-stroke span) was similar, particularly when greater precision was required. Another more recent study with singers (Chitalkina et al., 2021), reported that processing musical incongruities affects anticipation mechanisms (eye-time span), and changes in pupil size indicate less processing difficulty in singing compared to playing from notation.

In terms of expertise, expert musicians exhibit superior decoding capabilities. This is reflected in fewer and shorter fixations, larger EHS, and larger saccade sizes (Gilman and Underwood, 2003; Drai-Zerbib and Baccino, 2005; Penttinen et al., 2015). They also demonstrate superior perceptual skills (Sheridan et al., 2020) and enhanced cross-modal competences (Drai-Zerbib et al., 2012). Moreover, they show more efficient establishment and use of retrieval structures (Williamon et al., 2002; Perra et al., 2022) and the capacity to use auditory imagery during the reading process (Lee, 2006). Expert musicians process music stimuli more effectively as “chunks” or groups of notes (Kinsler and Carpenter, 1995; Polanka, 1995; Waters et al., 1997) and integrate musical information with fewer regressive saccades (Drai-Zerbib and Baccino, 2005; Penttinen et al., 2013).

To our knowledge, and considering Puurtinen's review (Puurtinen, 2018), two studies on EHS in flute performers are available. Schmidt (1981), reported inconclusive results, while Thompson (1987) found a positive association between sight-reading abilities and eye performance span in a study with 30 flutists. Research into EHS, specifically the relationship between anticipation mechanisms and music structure or the effect of practice on EHS, has received limited attention, with few publications (see Perra et al., 2021 for a review). To our knowledge, no other work has explored the connection between EHS in music reading and performers' respiratory mechanisms, assuming the involvement of the body in anticipation processes from the perspective of embodied musical cognition.

Breathing and air column management (air flow and pressure) during performance on a wind instrument are fundamental skills, necessary for interpreting music of various styles and complexities, which must be carried out with a certain degree of voluntary control over breathing (Faske, 2013; Woodberry et al., 2016). However, despite the emphasis placed by many pedagogues on the importance of proper breathing during musical performance, there is no consensus or evidence regarding the optimal approach to breathing (Ackermann et al., 2014). Although breathing is an automatic process controlled by the respiratory center of the nervous system, the frequency, depth, and rhythm of breathing can be unconsciously modified by mental emotions or consciously by different breathing patterns (Kochman et al., 2011). Moreover, the elastic recoil pressure of the respiratory system varies with lung volume (Cossette et al., 2000).

In the case of the flute, considered a low-pressure instrument (Brown and Thomas, 1990), its execution with high lung volume is compensated by antagonistic activity of inspiratory muscles (Cossette et al., 2010a). Respiratory support contributes to the antagonistic contraction of non-diaphragmatic inspiratory muscles, allowing the maintenance of high lung volume and control of pressure variations in the embouchure necessary for high-quality performance (Cossette et al., 2008). Vauthrin et al. (2015) observed in a case study, and through optoelectronic plethysmography, that lung volume patterns vary depending on the type of task (repertoire pieces and musical scales); however, the relationship between music structure and control parameters (embouchure pressure, lip opening area, lip-to-edge distance) is dynamic and intervenes independently of lung volume (Cossette et al., 2010b). Additionally, Vauthrin (2015) reported correlations between musical phrase duration, inspired volume, and work performed during inspiration. Furthermore, professional flute players use varied strategies and muscle groups in different sequences when playing the same note at a similar intensity (Cossette et al., 2000).

Turning to other musical instruments, de Manzano et al. (2010) studied 21 pianists' fluency in the classical-romantic repertoire, using physiological measures like piezoelectric breath sensors. They found that fluency correlated with blood pressure, heart rate, and breathing depth, indicating the involvement of emotional and attentional systems in musical fluency (significant effect of breathing depth on increasing fluency). Several studies indicate that musicians regulate their breathing in response to musical structure. Sakaguchi and Aiba (2016) found that pianists' breathing patterns are influenced by musical characteristics such as tempo and dynamics. In a pilot study, King (2017), found that pianists' breathing patterns varied with different pieces and according to musical gestures, structural elements, tempo, and physical movement. Similarly, Igarashi et al. (2002) used a circumferential breathing sensor to measure the effect of breathing on cellists' interpretation. They demonstrated through a computational algorithm that musicians tend to breathe at the same position during repeated musical passages, suggesting that breathing is controlled in response to musical structure.

Research indicates that pupil motility serves as an index of cognitive load and arousal (Granholm et al., 1996; Holmqvist et al., 2011; Price et al., 2013) and is related to the noradrenergic system (Strauch et al., 2022; Fink et al., 2024). Pupil diameter increases with higher cognitive load (Weber et al., 2015) and exciting stimuli (Bradley et al., 2008), but decreases when the cognitive demands exceed processing capacity (Granholm et al., 1996). Schaefer et al.'s (2023) review highlights the lack of consensus regarding the relationship between respiration and pupil dynamics, suggesting that this association may become clearer when respiratory parameters are evaluated individually (e.g., breathing rate, breathing depth). However, there is general agreement that arousal significantly affects both pupil dynamics and respiration (Onorati et al., 2013). While most music-related studies focus on passive listening, Vidal et al. (2024) found that pupil dilation increased under different conditions: no movement (only music listening), body sway to music, singing without body sway, and body sway plus singing. Pupil dilation was greater during the conditions involving movement, particularly when combined with singing, indicating that cholinergic-associated pupil activity reveals arousal levels. There is limited research on pupil responses during music reading: Hadley et al. (2018) found an increase in pupil diameter when processing chords with altered resolution notes, while insights may be obtained from the study by Chitalkina et al. (2021) cited above. To our knowledge, the relationship between pupil dilation and respiratory mechanisms during a written music performance task has not been directly investigated.

The aim of this research is to investigate the relationship between EHS, respiratory mechanisms, and eye movement measures in musicians learning a new flute piece. Specifically, we examine how the related variables are influenced by musicians' expertise, musical structure, practice, and learning strategies. A key focus is on the pupillary response during musical reading and its connection to EHS and respiratory mechanisms.

Hypothesis 1: Considering a multivariate perspective, we expect anticipation and breathing variables to be well represented in the factorial plane and associated with expertise. Skilled musicians should exhibit enhanced anticipation abilities, enabling more efficient adaptation of breathing patterns, including breathing rate, amplitude, and thoracic-abdominal balance. See Kochman et al. (2011) for an example of this adaptation.

Hypothesis 2: Grounded in embodied musical cognition (Leman et al., 2017), perceiving musical structures and emotional responses to music involves adapting breathing patterns as part of the meaning-making process. This adaptation enables instrumentalists to update their performance continuously, constructing meaning during interpretation (Assinnato and Blas Pérez, 2013). We expect that anticipation mechanisms and breathing patterns will vary based on music structure. This also applies to eye movement measures. In this context, we look for an increase in fixation durations and pupil dilation, and a decrease in anticipation variables with more complex phrases. Breathing patterns should adapt to musical structure and dynamics, as indicated by prior studies (Laczika et al., 2013; Vauthrin, 2015; Vauthrin et al., 2015).

Hypothesis 3: With practice (repeated executions), we anticipate modifications compared to the sight-reading phase. Specifically, the expected changes include: (1) Breathing patterns: an increase in the amplitude of thoracic and abdominal movements, along with stabilization of the sound pressure level, consistent with findings in opera singers (Foulds-Elliott et al., 2000); (2) Anticipation: an increase in EHS with practice, based on previous research outcomes (as discussed in the Introduction); and (3) Eye movement measures: a decrease in the number and duration of fixations.

## 2 Methods

### 2.1 Participants

The study included 27 flutists (13 females) from Valparaíso and Santiago, Chile, aged 19–42 years (*M* = 27.6, *SD* = 6.29). Expertise groups included 13 professional flutists and 14 undergraduates in their final 4 years of conservatory studies. Participants' musical backgrounds were assessed through self-reports on years of flute playing (*M* = 14.96, *SD* = 6.36) and previous experience in music reading and performance. Their height ranged from 1.54 to 1.84 m (*M* = 1.68, *SD* = 0.09). No participant reported being familiar with or having previously played the piece. All received compensation and provided informed consent.

### 2.2 Stimulus

**Figure 1:**
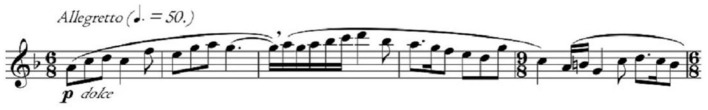
Excerpt featuring the initial five measures from the second movement of Koechlin's sonata for flute.

Musicians performed an excerpt from the second movement of Koechlin's Sonata for Flute and Piano, Op. 52 (Salabert Editions), consisting of 22 measures grouped into four phrases: measures 1–7, 7–12, 13–18, and 18–22. French notations on the score were omitted (see Figure 1). The music score was formatted as a JPEG with dimensions of 1,148 × 1,080 px and a resolution of 300 px/inch. The average distance between semiquavers was ~0.17 cm, with a bar width of 1.85 cm and a bar height of 0.33 cm. Consult the Supplementary Figure 1 for the complete score.

Four professional flutists rated each phrase of the piece from 1 to 10, considering the difficulty in learning the piece in a short session for a conservatory graduate (score 1) and another professional (score 2). The averaged scores for phrases 1 and 2 were (2) and (2.75), respectively, while phrases 3 and 4 scored (2.5) and (2.25) respectively.

### 2.3 Apparatus

For the recording of eye movements, a Tobii TX300 eye-tracker was used, operating at a sampling rate of 250Hz and connected to a computer equipped with Tobii Pro Lab 1.2 software. The distance between the flutist's head and the screen was ~600 mm. Flutists were recorded using a Sennheiser e914 microphone connected to a PreSonus StudioLive 16.0.2 digital recorder. Respiratory measurements were obtained using sensors (SA9311M; Thought Technology), connected to a ProComp 5 Infiniti system unit (Thought Technology), allowing for the conversion of analog signals to digital (sampling rate = 256 Hz). Following the recommendation of de Manzano et al. (2010), two respiratory sensors were employed simultaneously to measure both thoracic and abdominal breathing. The illumination in the experiment was 120 lux, measured with a Mastech MS66120 lux meter placed at the participants' head position during the music reading task.

### 2.4 Design and procedure

The experiment was conducted at the Language and Cognition Lab, at the Pontificia Universidad Católica de Valparaíso. Participants completed a pre-questionnaire about their musical background. Sensors were placed around the abdomen and thoracic cavity of musicians, below the sternum or mammary gland level for female participants. A 9-point calibration phase preceded the presentation of the musical stimulus. Participants performed the piece four times, with an option for 2 min of practice between the first and second trials. Before the first sight-reading trial, musicians had 20 s to silently read the score. Musicians performed seated to minimize potential movement of artifacts, as thoracic and abdominal breathing patterns should vary with posture (Ackermann et al., 2014). We aimed to explore expertise nuances in music performance, focusing on technical demands across different expertise levels. The selected musical piece was designed to present low technical demands, confirmed by the evaluation of an expert panel. The study adhered to the Ethics Committee guidelines of the Pontificia Universidad Católica de Valparaíso.

### 2.5 Preprocessing data

A custom C++ application was developed to collect respiration data from the ProComp device through the manufacturer's APIs, facilitating synchronization with other devices. TTL signals from the eye tracker and the ProComp 5 decoder were recorded on a PreSonus StudioLive 16.0.2 digital recorder, alongside performance data, in audio (wav) files at a sampling rate of 44.1 kHz. A MATLAB script was employed to synchronize signals from the three devices, correcting a minor drift (~6 ticks/2,048 = 3 ms/min recording) in the ProComp5 hardware through linear regression (see Salit and Turk, 1998). The eye tracker exhibited no drift, the Tobii device automatically adjusting any variances between transmitted and received pulses.

The audio signal from all four performances was analyzed using a MATLAB application designed to convert flute audio from WAV to MIDI format. The process involved correcting DC offset, segmenting the audio into 10-ms frames for sound intensity and pitch detection via short-time autocorrelation. MIDI files were processed using the MIDI Toolbox (Eerola and Toiviainen, 2004), and output MIDI files were generated using KaraokeMidiJava.jar (Smit, 2011). To address inaccuracies in MIDI conversion, a MATLAB script was developed, incorporating elements of Dynamic Matching Performance to Notation (Large, 1993) for comparisons between MIDI-converted flute recordings and dynamically generated MIDI models based on musical stimuli and participant's tempo. Remaining errors were rectified manually using a MATLAB-based MIDI editor.

For pupil dilation, raw data was extracted using the Tobii I-VT (Fixation) filter (left pupil). A blink detection function was applied (Hershman et al., 2018), with a maximum allowable gap of 100 ms between two sequential sets of missing data. Outliers (data points more than three scaled MAD from the median) were removed for each trial. Subsequently, linear interpolation was performed using the MATLAB function interp1. A third-order IIR Butterworth bandpass filter with a cutoff frequency of 2 kHz was applied to smooth the data.

For respiratory data, a continuous wavelet transform spectrogram was generated from the raw respiratory signals extracted from ProComp5 to identify the frequencies to be avoided with the filtering of a third-order IIR Butterworth bandpass filter. A frequency range of 0.08 to 10 Hz (Sensor 1) and 0.1 Hz to 3 Hz (Sensor 2) was applied, following the recommendations of Sánchez-Solís et al. (2023). Peaks and valleys exceeding two standard deviations were excluded prior to min-max normalization.

### 2.6 Data analysis

MATLAB programming enabled computation of the EHS for each of the 116 notes in the score, based on the nearest fixation in temporal and/or spatial proximity, synchronized with both MIDI event and eye-tracker data. Two measures were calculated: (1) the eye-hand span in notes or semiquavers (EHSN), representing the smallest difference in notes between the performed and fixated note, and (2) the eye-hand span in terms of time (EHST) or “time-index,” denoting the temporal distance between these two events. With regard to EHSN, if a musician fixates on more than one point while playing, the farthest fixation is considered for the calculation. Fixations lasting longer than 2,000 ms were omitted from the dataset (Holmqvist et al., 2003). For error calculation, a mean score was computed for each musical phrase, incorporating additions, substitutions, and deletions.

Regarding respiration data, breathing time sequences and breathing cycles were identified using the SpeechBreathingToolbox (MacIntyre and Werner, 2023). Additionally, offset-onset MIDI notes and acoustic signals were utilized for breathing data calibration to further enhance the accuracy and precision of the data collected. A MATLAB application was employed to prepare the following assessed variables: (1) mean amplitude of abdominal and thoracic movement within musical phrases: this measures the average depth or intensity of breathing during musical phrases; (2) number and duration of breathing cycles within musical phrases: this provides insights into the rhythm and pacing of breathing during musical performance, which can impact the musical expression and quality; (3) breathing rate: this measures the number of breathing cycles per minute, providing an overall indication of the timing of breathing during the musical performance; (4) differences in thoracic and abdominal performance ratio: this evaluates the balance and coordination between thoracic and abdominal breathing.

This multi-faceted approach to analyzing respiratory data provides valuable insights into the breathing patterns and techniques employed by musicians during performance. It can be particularly useful for understanding the relationship between breathing and anticipation, as well as for assessing and enhancing respiratory efficiency and control in musical performance.

## 3 Results

Flutists displaying outliers exceeding two standard deviations from the mean in one or more musical phrases were excluded from the analysis, adhering to established guidelines (±2.5 SD from the mean) (Orr et al., 1991; Aguinis et al., 2013). Descriptive statistics are presented in Table 1.

**Table 1:** Performance, eye movement, anticipation, and respiratory measures: means, standard deviations, and confidence intervals.

**Measures**	***M***	***SD***	**95% CI**	
			***LL***	***UL***
Errors	9.32	6.07	6.81	11.83
SPL	0.40	0.11	0.39	0.41
Perf D	71.265	8.6	67.79	74.74
Fix N	35.59	9.75	34.65	36.53
Fix D	431.92	98.92	422.44	441.4
Pupil D	3.38	0.41	3.34	3.42
EHSN	2.09	0.38	2.05	2.13
EHST	0.96	0.23	0.93	0.97
Thoracic MA	0.38	0.07	0.37	0.38
Abdom MA	0.38	0.07	0.37	0.39
BreathCycle N	2.91	0.99	2.82	3.01
BreathCycle D	6.49	1.91	6.30	6.68
Breath rate	9.45	2.78	9.19	9.72
Th-A ratio	1.9	1.88	1.72	2.07

CI, confidence interval; SPL, sound pressure level in normalized dB; Perf D, performance duration (in s); Fix N, number of fixations; Fix D, fixation duration (in ms); Pupil D, pupil dilation (in mm); EHSN, eye-hand span in notes (semiquavers); EHST, eye-hand span in time or time-index; Thoracic MA, normalized thoracic movement amplitude; Abdominal MA, normalized abdominal movement amplitude; BreathCycle N, number of breathing cycles; BreathCycle D, duration of breathing cycles (in s); Th-A ratio, differences in thoracic and abdominal performance ratio.

### 3.1 Principal component analysis

The average score across all four trials was computed for the following variables: performance measures (errors, performance duration and sound pressure level); eye movement measures (pupil dilation, fixation count and fixation duration); anticipation measures (EHSN and EHST); respiratory measures (mean abdominal and thoracic breathing amplitude within musical phrases, number and duration of breathing cycles within musical phrases, breathing rate, mean differences in thoracic and abdominal performance ratio within musical phrases).

Based on their representation on the factorial plane, the following variables were selected: EHSN, EHST, thoracic movement amplitude (Thoracic MA), abdominal movement amplitude (Abdominal MA), pupil dilation (Pupil D). Supplementary variables included performance duration, due to its influence on other eye-movement measures reported in the literature, as well as fixation duration, number of breathing cycles and differences in thoracic and abdominal performance ratio (as it was a compound variable; see Table 2).

**Table 2:** Correlation matrix.

	**1**	**2**	**3**	**4**	**5**	**6**	**7**	**8**	**9**
1. EHSN	1	0.75^***^	0.53^**^	0.23	0.13	−0.35	−0.33	0.02	−0.61^***^
2. EHST	0.75^***^	1	0.34	0.06	−0.04	−0.03	0.32	0.25	−0.60^***^
3. Thoracic MA	0.53^**^	0.34	1	0.42^*^	0.40^*^	−0.52^**^	−0.31	−0.34	−0.21
4. Abdominal MA	0.23	0.06	0.42^*^	1	0.36	−0.14	−0.27	−0.20	0.19
5. Pupil D	0.13	−0.04	0.40^*^	0.36	1.00	0.02	−0.23	−0.30	0.17
*6. Fix D*	−0.35	−0.03	−0.52^**^	−0.14	0.02	1	0.42^*^	0.29	0.19
*7. Perf D*	−0.33	0.32	−0.31	−0.27	−0.23	0.42^*^	1	0.36	−0.07
*8. BreathCycle N*	0.02	0.25	−0.34	−0.20	−0.30	0.29	0.36	1	−0.09
*9. Th-A ratio*	−0.61^***^	−0.60^***^	−0.21	0.19	0.17	0.19	−0.07	−0.09	1

Supplementary variables are indicated in italics.

EHSN, eye-hand span in quavers; EHST, eye-hand span in time or time-index; Thoracic MA, thoracic movement amplitude; Abdominal MA, abdominal movement amplitude; Pupil D, pupil dilation; Fix D, fixation duration; Perf D, performance duration; BreathCycle N, number of breathing cycles; Th-A ratio, differences in thoracic and abdominal performance ratio.

^*^p < 0.05.

^**^p < 0.01.

^***^p < 0.001.

**Figure 2:**
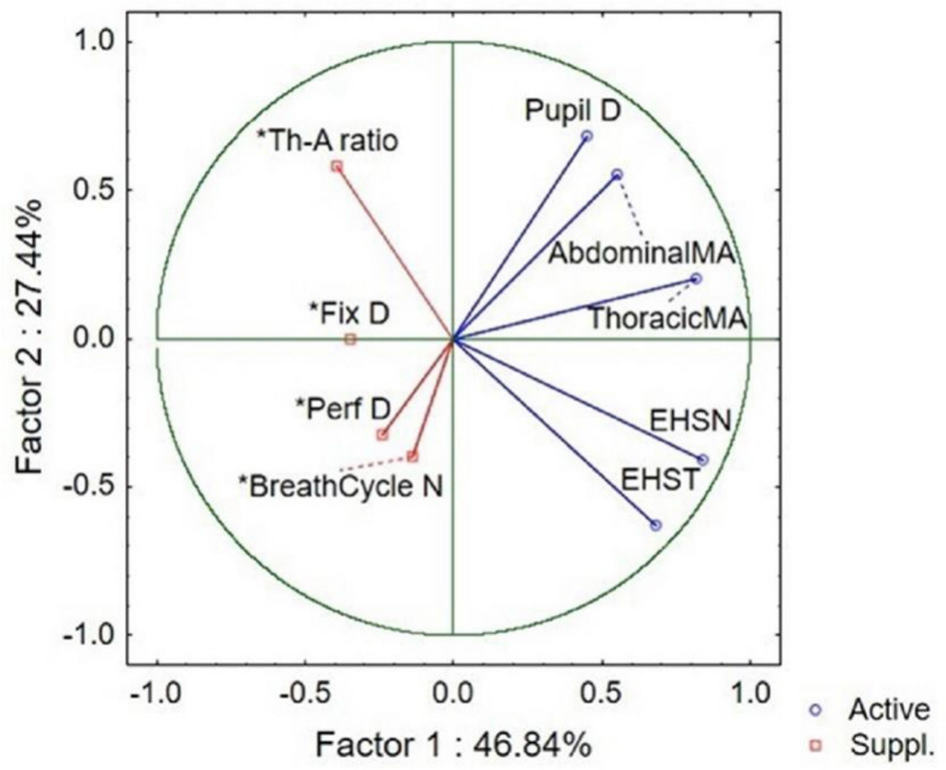
Principal component analysis: the length of each vector indicates the variable's contribution to establishing the principal components. Supplementary variables are indicated by the (*) symbol.

Two principal axes were observed: the first related to anticipation (EHSN and EHST) and the second to thoracic and abdominal movement amplitude (Thoracic MA and Abdominal MA). Between these axes, there is relative independence except for the thoracic movement amplitude, which shows a positive correlation with the anticipation variables, highlighting the complexity of the respiratory techniques implemented by performers during interpretation of the musical piece. Differences in the thoracic and abdominal performance ratio are negatively correlated with EHS (see Figure 2).

The interpretation of pupil dilation presents challenges in our analysis. While its inclusion as an active variable may diminish the variance explanation of the first principal component, its correlation with thoracic movement amplitude could enrich its interpretation. Additionally, performance variables shifted the barycenter in the analysis, likely due to a significant decrease in error rates from the second performance onward [*F*_(3, 75)_ = 12.93, *p* < 0.001, η*p*^2^ = 0.33]. A similar change occurred in the number and duration of breathing cycles, for this reason we have chosen to include them as supplementary variables and to discuss their implications in relation to the active variables. Given the limited representation of performance duration in the factorial plane, it will also be treated as a supplementary variable.

Remarkably, the variables of interest in the study are strongly represented on the factorial plane. This noteworthy finding provides methodological evidence for delving deeper into these variables in the subsequent section.

### 3.2 Anticipation and breathing

Based on the results obtained from the PCA, we analyze the effects of musical structure, practice, and expertise on the variables of interest. To do this, we conduct a 4 × 4 × 2 repeated measures ANOVA for each variable separately, with factors of musical structure or phrases, trials, and skill levels, respectively. A Bonferroni adjustment (0.055/4) is applied to control for an overall type I error. Two flutists were removed from the EHSN, EHST, thoracic sensor analysis and abdominal sensor analysis; one participant was excluded from the pupillary dilation analyses (see Table 3).

**Table 3:** Repeated measures ANOVA summary for eye movement, anticipation and breathing measures.

**Measures**	***n***	***df***	**Musical phrase**		***df***	**Trial**		***df***	**Expertise**	
			***F-*value**	***ηp* ^2^**		***F*-value**	***ηp* ^2^**		***F*-value**	***ηp* ^2^**
EHSN	25	3.69	160.06^**^	0.411	3.69	3.93^*^	0.146	1.23	7.01^*^	0.233
EHST	25	3.69	18.33^**^	0.443	3.69	0.62	0.026	1.23	0.65	0.027
Thoracic MA	25	3.69	43.67^**^	0.655	3.69	0.39	0.016	1.23	1.18	0.049
Abdominal MA	25	3.69	29.83^**^	0.564	3.69	0.61	0.029	1.23	2.65	0.103
Pupil D	26	3.72	133.56^**^	0.848	3.72	11.19^**^	0.318	1.24	0.003	0.001

d.f., degrees of freedom; EHSN, eye-hand span in quavers; EHST, eye-hand span in time or time-index; Thoracic MA, thoracic movement amplitude; Abdominal MA, abdominal movement amplitude; Pupil D, pupil dilation.

^*^p <0.01.

^**^p <0.001.

#### 3.2.1 Musical structure

We observe a musical structure effect across all relevant variables (see Table 3). EHSN shows increased anticipation in the first phrase, alternating in subsequent phrases with the most significant difference between the first and second. The time-index increases gradually toward the third phrase and then declines. Both measures peak in the third phrase, which incorporates elements from previous phrases and introduces a new motif.

**Figure 3:**
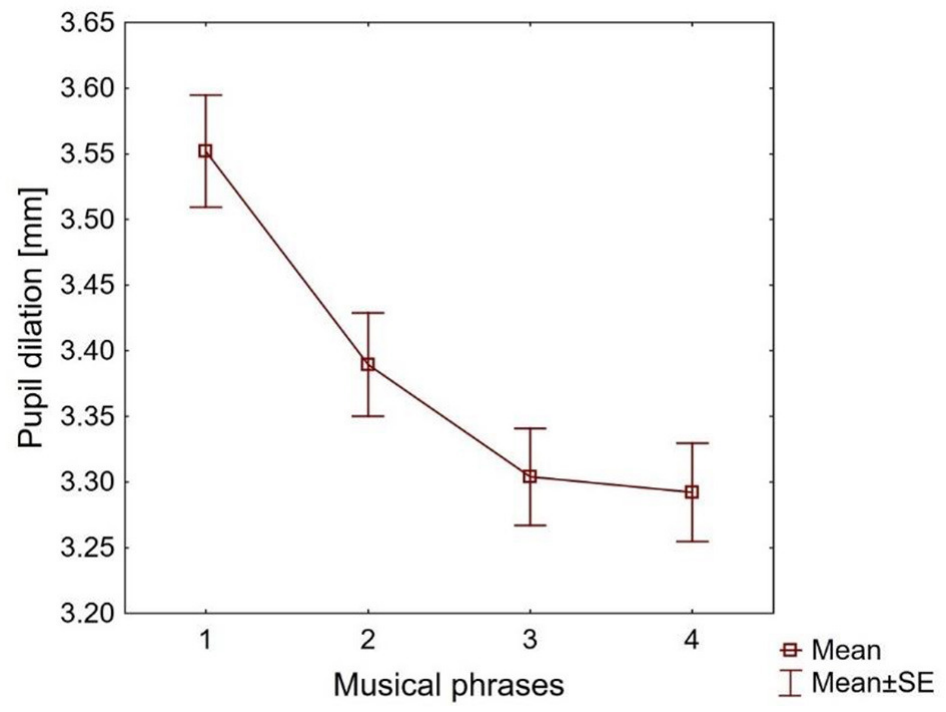
Variations in pupil dilation throughout the musical phrases. Error bars show the standard error of the mean.

Thoracic and abdominal movement amplitude present a similar profile to EHS but more gradual. A greater amplitude is observed in the first phrases, which then declines and begins to rise slowly again without reaching the initial values. This aligns with the composer's indications in the score, showing no significant dynamic contrasts as it does not exceed the indication “piano.” Regarding pupil dilation, a gradual decrease throughout the phrases was observed (see Figure 3 and Table 4).

**Table 4:** Mean values and probabilities of musical phrase and trial factors for each measure, analyzed using Tukey's Honest Significant Difference (HSD) test.

**Measures**	**Phrase 1**	**Phrase 2**	**Phrase 3**	**Phrase 4**	**Trial 1**	**Trial 2**	**Trial 3**	**Trial 4**
EHSN	2.23 a (0.37)	1.97 c (0.38)	2.11 b (0.39)	2.04 bc^**^ (0.35)	1.99 a (0.34)	2.10 ab (0.42)	2.16 b (0.37)	2.10 ab^*^ (0.39)
EHST	0.89 c (0.19)	0.97 b (0.25)	1.05 a (0.25)	0.92 bc^**^ (0.20)	0.94 (0.19)	0.95 (0.23)	0.97 (0.24)	0.96 *ns* (0.26)
Thoracic MA	0.37 c (0.07)	0.37 c (0.06)	0.38 b (0.07)	0.39 a^**^ (0.06)	0.37 (0.09)	0.38 (0.05)	0.38 (0.06)	0.37 *ns* (0.06)
Abdominal MA	0.37 c (0.10)	0.37 b (0.10)	0.38 b (0.10)	0.39 a^**^ (0.10)	0.39 (0.11)	0.37 (0.11)	0.37 (0.09)	0.36 *ns* (0.10)
Pupil D	3.55 a (0.44)	3.39 b (0.4)	3.30 c (0.38)	3.29 c^**^ (0.38)	3.4 a (0.4)	3.42 a (0.43)	3.37 b (0.41)	3.35 b^**^ (0.40)

Groups are denoted by letters (a, b, c, ab or bc) based on the results of the ANOVA Tukey test. Letters distinguish significant differences between groups within musical phrases or trials. If no significant difference was found, no letter is assigned. Standard deviations (*SD*s) are presented in parentheses.

EHSN, eye-hand span in quavers; EHST, eye-hand span in time or time-index; Thoracic MA, thoracic movement amplitude; Abdominal MA, abdominal movement amplitude; Pupil D, pupil dilation.

^*^p <0.01.

^**^p <0.001.

#### 3.2.2 Practice effect

We observed an effect of the trial on both EHSN and pupil dilation. The learning curves differ between these two variables, with EHSN showing a peak in the third trial and a slight decrease in the last trial. In contrast, pupil dilation tends to decrease in the third and fourth trials (see Table 4). It is important to consider that the piece's difficulty could influence the effects observed; overall, the flutists were able to fluently read the piece at the tempo indicated by the composer from the first reading. Indeed, no significant differences were observed in the performance duration across the trials *F*_(3, 69)_ = 2.52, *p* = 0.06, η*p*^2^ = 0.099.

#### 3.2.3 Expertise

**Figure 4:**
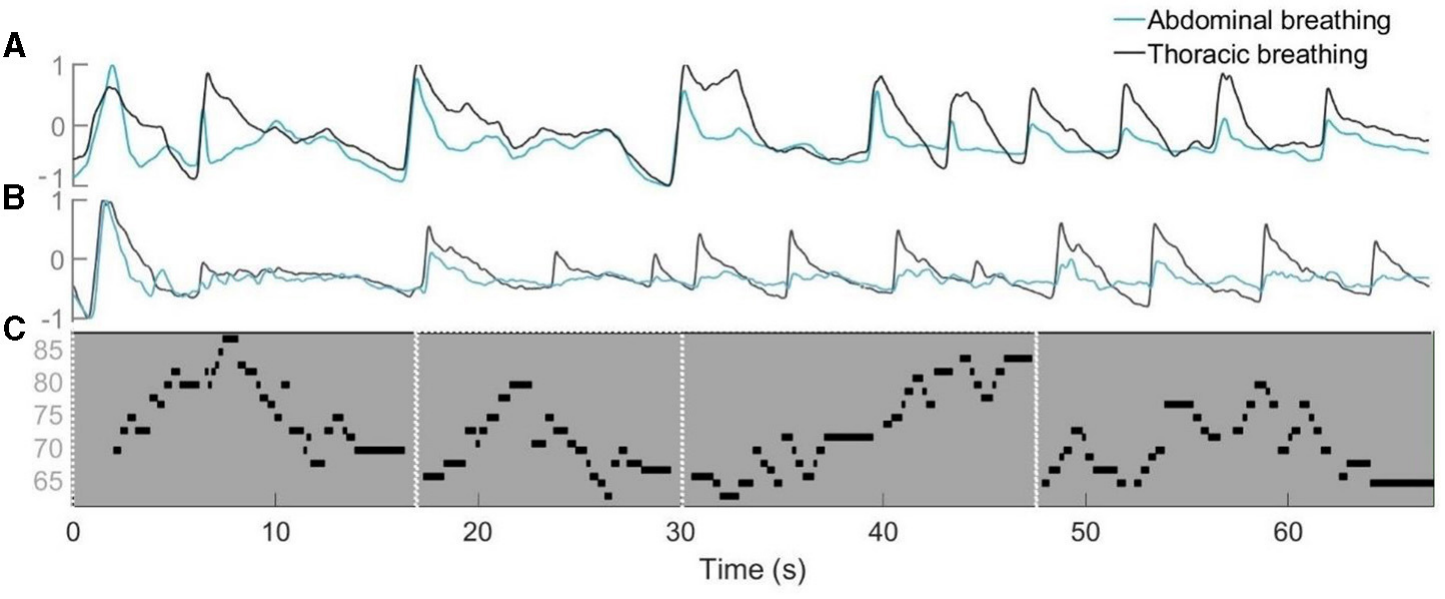
Respiratory signal of an undergraduate musician **(A)** compared to that of an expert musician **(B)**. The bottom section shows a piano roll representing MIDI notes **(C)**. Vertical dotted lines indicate the boundaries of each musical phrase.

In our research, we examined two approaches to musical expertise: the ability to play without errors (examined in the previous section with the PCA) and the formal training of musicians. We found a significant effect of formal expertise only in the EHSN. Additionally, expertise-phrase interactions in the EHSN [*F*_(3, 69)_ = 3.63, *p* = 0.02, η*p*^2^ = 0.136] highlight that differences are detectable between more and less experienced musicians, especially in the third musical phrase where a new musical motif is introduced (see Figure 4 and Table 3).

## 4 Discussion and conclusions

In this study, we focused on investigating the cognitive processes and physiological responses involved in learning a piece of music for flute among musicians of varying levels of expertise. We assessed anticipation and respiratory variables in relation to musical structure, practice, and expertise. Consistent with previous findings in advanced pianists (see Cara, 2018, 2023), we observed an effect of musical structure on all the variables analyzed, confirming our second hypothesis. Specifically, we identified an association between anticipation and thoracic movement amplitude, suggesting the existence of a coupling between anticipation and breathing from the early stages of learning. Due to the discrete technical challenges of the piece, a clear expertise effect was not evident, raising questions about the flexibility required to study expertise and partially refuting our first hypothesis. Furthermore, pupil dilation was associated with abdominal movement amplitude, indicating an embodied emotional or cognitive component linked with music structure processing. We believe we have contributed to a better understanding of short-term learning as an embodied phenomenon, which we will discuss further.

### 4.1 Coupling of anticipation and breathing

When viewed from an embodied perspective, anticipation involves understanding musical performance as a dynamic process, where action is anticipatory and the cognitive or motor capabilities of expression are underpinned by anticipation (Schulkin, 2013). This embodied expression holds significant importance for wind instrument players as it is directly linked to respiratory mechanisms. In the present study, we demonstrated an association between respiratory mechanisms and anticipation in terms of the number of notes (EHSN) and time (EHST), which is supported by a high representativeness in the factorial plane compared to other variables. This finding confirms our second hypothesis and extends its scope. Indeed, we observed an adaptation of both breathing patterns and anticipation to the musical structure; as previously mentioned, this adaptation occurs in a coupling between the two variables.

Furthermore, breathing patterns adapt to the dynamics of the score, confirming observations in previous studies (Laczika et al., 2013; Vauthrin, 2015; Vauthrin et al., 2015), which is in line with our second hypothesis. The coupling of anticipation and breathing persists throughout the various reprises, highlighting the importance of managing interoceptive information in musicians as part of an expertise and embodiment phenomenon. Indeed, there is evidence that interoceptive bodily signals have an impact on voluntary movements and that these movements could be preferably planned during expiration (Park et al., 2020). Moreover, this interoceptive information is important in various sensory processes (Park et al., 2014), including emotional processing (Garfinkel et al., 2014).

In relation to our findings, it is possible that increasing thoracic or abdominal expansion facilitates enhanced interoceptive information processing during the expiratory phase. This may relate to the use of different kinematic strategies. It has been observed in lyrical singers that these kinematic strategies are partly determined by the context (Foulds-Elliott et al., 2000) and also by the emotional connection to the task (Pettersen and Bjørkøy, 2009; Kochman et al., 2011). This emotional and cognitive component could guide the interpretation of the results of our PCA, particularly concerning the connection between thoracic movement amplitude and pupil dilation. Indeed, pupil dilation significantly decreases across the trials and phrases which is consistent to some extent with the literature consulted (Chitalkina et al., 2021; Vidal et al., 2024). Conversely, the EHS tends to increase across the trials, while the EHST remains constant at ~1 s, supporting findings from previous studies (see Introduction). Considering evidence regarding the coordination between respiration and oculomotor behavior (Rassler and Raabe, 2003) and the potential for breathing patterns to reduce cognitive load (Varga and Heck, 2017), the dynamic relationship between anticipation and breathing mentioned above appears to re-emerge. It should be noted that our results for pupil dilation differ somewhat from studies conducted on non-musical participants evaluating music predictability and learning (i.e., Bianco et al., 2019). However, they corroborate our hypothesis regarding adaptation to musical structure and its variation across the trials. Additionally, our findings align with previous studies with musicians performing music for string quartets, where an influence of movement and musical complexity on pupil size was observed (Bishop et al., 2021). Similar results were reported by Vidal et al. (2024), who noted increased pupil dilation in musicians while singing and moving (see Section 1).

An alternative approach to analyzing our findings in relation to PCA is to consider the observed contribution of breathing patterns (thoracic and abdominal movement amplitude) to the principal components. Indeed, as reported by Collyer et al. (2008) and Collyer et al. (2009) in opera singers, various possible contributions of the abdomen and rib cage to lung volume exist during performance, either simultaneously, sequentially, or in combination. These patterns vary among performers (Cossette et al., 2000). In our study, we observed that musicians employ different breathing patterns, some being contrasting, which is reflected to some extent in the factorial plane. These differences could primarily be attributed to the different training schools of the flutists, each with distinct support and breathing techniques. These observations are partly supported by supplementary variables, particularly the compound variable mean differences in thoracic and abdominal performance ratio within musical phrases, which highlights a particular dominance of abdominal breathing (in some musicians). This variable shows a strong negative correlation with the EHS (*r* = −0.61) and is not particularly associated with expertise. Consequently, effective anticipation should be related to a balanced and coordinated breathing pattern, which might be aligned with deeper abdominal breathing. This is in line with the findings of Sehmann (2000) regarding the potential for consciously improving abdominal breathing within a relatively short period. Furthermore, the control of voluntary breathing involves the coordination of thoracic and abdominal breathing, as noted by Higashino et al. (2022).

The literature also mentions the existence of thoraco-abdominal asynchrony more associated with certain pathologies or respiratory dysfunctions (Sackner et al., 1984; Hammer and Newth, 2009; Porras et al., 2017; Higashino et al., 2022). It is relevant to note that one of the participating musicians reported having contracted SARS-CoV-2 on five occasions, as a result of which he perceived a reduction in his respiratory capacity. Upon concluding the protocol, we observed significant differences in the contribution of abdominal breathing, corroborated by measuring vital capacity at the end of the experiment. This aligns with the observations of Higashino et al. (2022), suggesting that an increase in respiratory frequency could lead to a shift toward costal breathing (which normally should not occur in wind instrument performers). We chose not to exclude this participant from the sample and preferred to report what was observed. Indeed, nearly a third of the participants reported having had COVID-19, and many others may have had it asymptomatically.

### 4.2 Music structure, practice and expertise

The effect of practice on EHS confirms our second hypothesis and aligns with prior research (Burman and Booth, 2009; Cara, 2023). As stated in the introduction, the impact of practice on EHS has not been systematically measured, and there are limited studies with various approaches. From our perspective, previous knowledge of the composer's style and the piece's difficulty, along with the musician's capacity to mobilize cognitive skills, appear critical for EHS improvement through practice. Our findings suggest that the embodied learning component linked to breathing management also significantly influences EHS. Pupil dilation significantly decreases with practice, a phenomenon not previously addressed in musical reading studies. This could relate to reduced cognitive load and the involvement of the attentional component across repetitions (Strauch et al., 2022), affecting pupil dilation and potentially confirmed by increased anticipation. The emotional component linked to pupil dilation is harder to establish but may relate to PCA observations. Pupil dilation correlates with thoracic movement amplitude and contributes to the PCA's second component along with the abdominal movement amplitude. In both interpretations, as posited by Varga and Heck (2017), it becomes apparent that the body plays a crucial role in influencing cognitive processes and breathing patterns.

We observed that more skilled musicians show independence from written notation, enhancing anticipatory processes in line with the observations of Drai-Zerbib et al. (2012). This partly confirms our first hypothesis, as the anticipated expertise effect in thoracic or abdominal movement amplitude was not observed. Moreover, the musicians' ability to play with fewer errors does not fully account for the dynamics of the coupling between anticipation and breathing, as confirmed by the PCA results. In relation to the duration and number of breathing cycles, we observed a broad range of patterns; however, no significant effects of expertise were identified. Notably, some musicians, particularly students, performed the piece with more or fewer breathing cycles than indicated in the score with phrase markings (*n* = 11). These patterns remained relatively consistent across performances, reflecting varied stylistic and technical approaches to the piece. In line with Kochman et al. (2011), who noted the adaptation of lyrical singers' respiratory volume according to the condition (rehearsal vs. performance), we could affirm that in our study, musicians adapt their breathing patterns according to the musical structure, but the effect is not significant across trials, partially refuting our third hypothesis. Furthermore, we observed a decrease in breathing amplitude following the first phrase. While this could reflect an adaptation to the dynamics specified by the composer, the interaction between phrase and reprise reveals that, despite varying breathing patterns within each phrase across the trials, the amplitude of the first phrase consistently remains higher. This suggests that certain breathing patterns are voluntary, while others are more flexible and involuntary, supporting the literature consulted (Pettersen and Bjørkøy, 2009). Additionally, the musical material evolves throughout the piece, with each musical phrase introducing new motifs while also incorporating elements from earlier phrases. The observed peak in the third phrase for both the EHS and time index is consistent with previous research in pianists (Cara, 2018, 2023).

In comparison to previous studies in woodwind players, our fixation durations are shorter on average than those reported by Zhukov et al. (2019). This discrepancy is probably due to the piece's complexity and the musicians' skill level. Indeed, ample evidence suggests that fixation duration serves as an indicator of expertise (Drai-Zerbib et al., 2012; Perra et al., 2022).

As a limitation of this study, forming expertise groups was challenging due to limited sample access, the advanced skill level of senior cycle students and the variability of musicians' profiles. Consequently, the lack of a clear expertise effect in many of the variables studied is not surprising, which aligns with previous research in pianists (Cara, 2018, 2023). We also aimed to address expertise in terms of the musicians' ability to play with few errors; however, the latter showed limited representativeness in the factorial analysis. This highlights the need to further investigate the role of expertise from various perspectives, including computational modeling, as consistent with recent literature on expertise in musical reading (see Perra et al., 2022). Other limitations of the present study concern the potential impact of COVID-19 on the participants' respiratory capabilities. Although we inquired about any history of COVID-19 among the flutists, it was challenging to precisely quantify the extent of this impact, especially considering that some might have had COVID-19 without being aware of it. Additionally, we would have liked to further control for variables that could affect respiratory capabilities, such as age, race, gender, and height. However, as previously mentioned, the specificity and size of our sample limited our possibility to do so. We also chose not to use a single musical instrument for all participants to avoid potentially disrupting many musicians and affecting their performance. Finally, it is worth mentioning the potential for pupil foreshortening error due to the musicians' movements while playing (Hayes and Petrov, 2016; Mathôt et al., 2018; Petersch and Dierkes, 2022). To counter this, we rigorously followed the preprocessing of the pupil signal.

From an educational perspective, incorporating body and emotional awareness into breathing techniques could be helpful for enhancing self-regulation. Additionally, the findings on pupil dilation emphasize the importance of focusing on the early stages of learning, where emotional and attentional components are integrated from the beginning. Considering these insights, fostering coordination between thoracic and abdominal breathing and emphasizing deep abdominal breathing alongside sight-reading tasks could be beneficial. Our study's results suggest that such integration may also influence the effectiveness of anticipation in music reading. Moreover, our analysis underscores the critical role of musical structure in the dynamic integration of cognitive and physiological processes, suggesting that the coupling between anticipation and breathing reflects the implementation of adaptive learning strategies tailored to musical phrases and trials.

It is important to interpret the conclusions of this study within the context of the specific participants and conditions used. As the study focused on a certain musical piece and instrument, results may differ if other musical material is used. Consequently, these findings should be viewed as specific to this study's context, and generalizations must be treated with caution.

Future directions of the research may explore the influence of biofeedback on performance, learning, and anticipation. Additionally, extending the use to different physiological signals could provide a more comprehensive view and allow for the development of feedback models. Likewise, another future research direction could focus on tailoring such biofeedback to the individual needs of each musician, potentially using artificial intelligence.

In summary, we investigated the relationship between anticipation, breathing, and performance in musicians of varying expertise levels learning a flute piece by Charles Koechlin. Our findings revealed a dual representation on the factorial plane: one dimension associated with performance planning, and another linked to embodied and emotional components. This supports a hypothesis of coupling between anticipation and breathing, emphasizing the intricate interplay between anticipation and embodied awareness in musical performance, and thus, as stated by Varga and Heck (2017), between body cognitive functioning and breathing. Additionally, the impact of musical structure on perceptual and cognitive processes emphasizes the critical role of contextual elements in music performance and learning.

## Data Availability

The raw data supporting the conclusions of this article will be made available by the authors, without undue reservation.
