# Supplementary figures and images for: Coupling of anticipation and breathing in expert flute performance: the influence of musical structure and practice
Source: Front Cognit. 2024 Sep 17;3:1425005. doi: 10.3389/fcogn.2024.1425005 (PMC13281148; doi:10.3389/fcogn.2024.1425005)

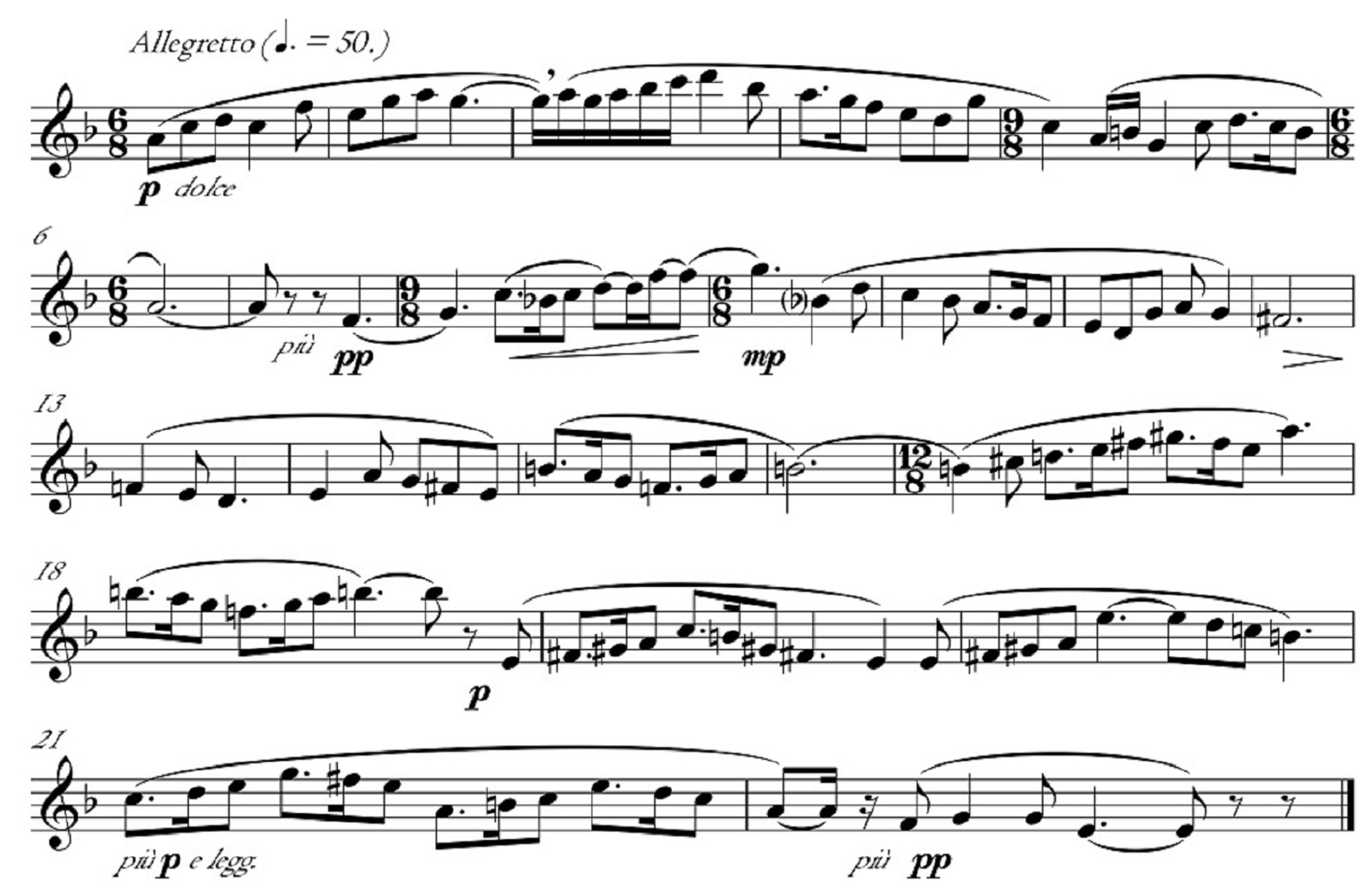

Supplement: Supplementary file 1 [file Image_1.jpeg]
